# The social tensions felt within: Explaining felt ambivalence about polarized societal debates through perceived opinion discrepancies in the social environment

**DOI:** 10.1111/bjso.12574

**Published:** 2022-09-11

**Authors:** Gonneke Marina Ton, Katherine Stroebe, Martijn van Zomeren

**Affiliations:** ^1^ Department of Social Psychology, Faculty of Behavioural and Social Sciences University of Groningen Groningen The Netherlands

**Keywords:** ambivalence, social discrepancy, social tension, societal debates

## Abstract

Within the context of polarized societal debates (e.g. abortion, racism, climate change), scholars often assume that individuals have clear‐cut positions, either in favour of or against the debated issue. However, recent work suggests that such debates can also be breeding grounds for felt ambivalence. Moving beyond previous work that mainly focused on ambivalence as internal cognitive conflict, we propose and test a social discrepancy hypothesis, which suggests that the discrepancies ambivalents perceive between and within their own opinion and the opinion of actors in their social network and society (e.g. friends, family, opinion‐based groups) positively explain their levels of felt ambivalence. In doing so, we quantitatively extend recent qualitative work by examining whether these social tensions are indeed felt within. To this end, we employed a multi‐survey research project (*N*s = 184, 181, 187) in the context of different societal debates in the Netherlands. Supporting our hypothesis across different debates, results showed that ambivalents' perceived opinion differences in the social environment explained their felt ambivalence. This suggests that polarized societal debates offer social discrepancies that, for ambivalents at least, can facilitate an internalization of social tensions.

## BACKGROUND

Polarized societal debates about social change, such as abortion, racism and climate change, are often characterized by two groups in favour or against the issue at hand. So far, social–psychological research has focused on how such dichotomous debates push or pull people into one of these groups with clear‐cut positions (Bliuc et al., 2021; Dunlap et al., 2016; Koudenburg & Kashima, 2021). However, we propose that these same social forces can make others feel *ambivalent* about the issue, as polarized societal debates emphasize the opinion differences between, but also within, relevant social and societal groups. In fact, recent work found that people with mixed feelings on the topic – ambivalents – may find themselves caught in the social crossfire of opposing positions held by social (e.g. friends, family members) or societal (e.g. proponents, opponents) groups (Ton et al., 2022 ). In other words, these individuals might experience ambivalence as they perceive such social discrepancies and feel these social tensions within.

Our line of thought fits with but also moves beyond theory and research on the experience of ambivalence (i.e. having mixed reactions, feeling torn and conflicted and/or being undecided; Priester & Petty, 1996; van Harreveld, van der Pligt, et al., 2009). In this work, felt ambivalence has long been associated with a discrepancy in *personal* beliefs and opinions (i.e. intrapersonal cognitive conflict). We build upon recent qualitative research revealing that, in the context of a polarized societal debate concerning racism in the Netherlands, ambivalents found themselves ‘caught in the social crossfire’ of opposing positions held by social forces (e.g. family, friends, opposing groups in society; Ton et al., 2022). In the current study, we aimed to corroborate and move beyond these findings by disentangling the different social forces that give rise to this experience and test the power of perceiving *social discrepancies*, defined as the presence of conflicting attitudes or beliefs about a social issue within social (e.g. friends, family members) or societal (e.g. proponents, opponents) groups in their social environment.

Our research aims to test the currently untested *social discrepancy hypothesis* within three different polarized societal debates in the Netherlands (i.e. about *Zwarte Piet*, fireworks and meat consumption). This hypothesis states that ambivalents' perception of social discrepancies in relevant social or societal groups positively explains felt ambivalence. Finding support for this hypothesis contributes to the literature by offering both a more generalizable and specific explanation of how social tensions in the context of societal debates can be felt within.

### Polarized societal debates as a breeding ground for felt ambivalence

Societal debates are social contexts in which there is a contentious debate about an issue that implies a potential social change in society (Ton et al., 2022). These societal debates are perceived to be polarized when the opinions in society are divided and opinion‐based groups (McGarty et al., 2009) form around these divisions (Koudenburg & Kashima, 2021). Such groups develop when individuals who share similar opinions form and identify with a social group that distinguishes itself from other groups by essentializing this opinion (Bliuc et al., 2007; McGarty et al., 2009).

Social–psychological research within such contexts typically focuses on the intergroup conflict between the opinion‐based groups. It assumes that stronger identification with these social groups induces stronger conformity to ingroup norms and more extreme opinions, leading to ideological and psychological distancing and ultimately (perceived) polarization between the different opinion‐based groups (Bliuc et al., 2021; Koudenburg & Kashima, 2021). Therefore, within that line of research, debates are often considered a tool of persuasion by those involved: a means to convince others to take a stance and act for social change.

However, such a focus on polarized societal debates may overlook the possibility that some individuals in these very same contexts may feel ambivalent. Indeed, polarization in societal debates often produces a choice situation with pressures to choose one of two sides, which makes the experience of ambivalence more likely (van Harreveld, van der Pligt, et al., 2009). Furthermore, societal debates can make visible that one's social network and groups are internally divided, which, we propose, makes the perception of social discrepancy and the experience of ambivalence on the topic more likely (Ton et al., 2022; see also Pillaud et al., 2013; van Harreveld, Rutjens, et al., 2009).

### The social discrepancy hypothesis

Previous research has more generally associated perceived discrepancy with the experience of ambivalence. Indeed within more cognitively oriented approaches, felt ambivalence is often considered the outcome of an intrapersonal conflict resulting from discrepancy: the results of simultaneously experiencing positive and negative attitudes about the same issue (e.g., Jonas et al., 2000; Newby‐Clark et al., 2002; Schneider & Schwarz, 2017; Thompson et al., 1995; van Harreveld et al., 2015). Furthermore, although discrepancies in individuals' attitudes are thought to be at the basis of feelings of ambivalence, feelings of ambivalence may only be present when people are made aware of the discrepancy, for example, when they have to make a decision (van Harreveld, Rutjens, et al., 2009; van Harreveld, van der Pligt, et al., 2009). Our analysis is compatible with previous work on the role of *personal discrepancies* in relation to ambivalence, but we move beyond this by focusing on ambivalents' perceived *social discrepancies* in the context of societal debates.

More specifically, initial evidence for this hypothesis came from interviews with Dutch participants who felt ambivalent in the context of a polarized societal debate about whether or not to retain the potentially racist character of *Zwarte Piet* (Black Pete; explained in more detail later in this article) in an annual Dutch tradition (Ton et al., 2022). The analysis revealed that ambivalent participants felt caught in the social crossfire of opinion differences between activist groups, between their friends and family, but also within groups they related to (e.g. their family was in favour while others were against). Although the ambivalence experienced by the participants seemed to reflect the social tension felt within, the study did not disentangle the different social forces at play in the context of societal debates and was limited by its reliance on a single polarized debate. The current work aims to follow‐up on this qualitative work by quantitatively disentangling social forces and testing the *social discrepancy hypothesis* within and across multiple polarized societal debates.

Other findings already indicate indirect support for our line of thought. For example, Priester and Petty (2001) found that *interpersonal* discrepancy between participants' attitudes and the perceived attitudes of positively regarded others (i.e. parents or other students) was predictive of felt ambivalence, above and beyond (intra)personal discrepancies. Additionally, correlational research on *contextual* sources of political ambivalence during election campaigns in the United States suggested that state differences in political ambivalence could be explained by the amount of competition between partisan campaigns in each state. Therefore, it argued that the salience of opinion differences could foster ambivalence in individual voters (Keele & Wolak, 2008). Such findings fit nicely with our suggestion that opinion differences in the social environment may be associated with the experience of ambivalence. We assume, however, that *social discrepancy* is particularly salient for ambivalents in polarized debates in which opinion differences are central and essentialized. In the current study, we follow up on these findings by comparing the different social tensions salient in polarized societal debates and test whether they explain felt ambivalence above and beyond personal discrepancy.

### The current research

We utilize a quantitative approach by collecting survey data about three different societal debates in the Netherlands. This methodological approach allows us to test whether previous qualitative findings (Ton et al., 2022) can be generalized to other societal debates and empirically differentiate which social conflict(s) people might find themselves caught in (i.e. between social or societal groups), hence *which* social tensions they feel within. Specifically, ambivalents might experience feelings of ambivalence because they perceive opinion differences between their personal opinion and that of significant others, between and within friends, family or groups that they belong to or between and within (activist) groups in society (Ton et al., 2022; Keele & Wolak, 2008; Priester & Petty, 2001). Moving beyond previous work, we test the *social discrepancy hypothesis*, which states that perceived opinion differences within or between social and societal groups positively explain ambivalents' felt ambivalence in the context of polarized societal debates.

We propose that polarized societal debates offer social forces that can give rise to *social discrepancy* and hence ambivalence. To avoid reliance on a single debate or issue (as in Ton et al., 2022), we tested the *social discrepancy hypothesis* in three contexts: the societal debates in the Netherlands concerning *Zwarte Piet* (also used in Ton et al., 2022), New Year's Eve fireworks and meat consumption. First, the *Zwarte Piet* debate in the Netherlands is a heated and recurrent debate about the national Sinterklaas (Saint Nicolas) tradition, in particular, due to the (potential) racism underlying the black‐faced assistant *Zwarte Piet*. *Zwarte Piet* has traditionally been portrayed by (White) adults wearing gaudy costumes, large golden earrings, afro‐style wigs, red lipstick and full blackface makeup. Second, the debate concerning the use of fireworks in the Netherlands on New Year's Eve is a recently recurrent discussion on the necessity of regulating or even prohibiting consumer fireworks. In contrast to most other countries, the Dutch New Year's Eve tradition is focused on the personal use of fireworks, which is largely unregulated and associated with many injuries and damages each year. Third, the debate surrounding meat consumption discusses the need to cut down on meat for environmental, health and animal welfare reasons. In contrast to the other two debates, this debate is not particular to the Dutch context. Indeed, there is ample research regarding the different motivations and positions related to vegetarianism (Rosenfeld, 2018; Ruby, 2012; Thomas et al., 2019).

## METHOD

Data were collected simultaneously for all three surveys in February 2020 (before Covid measures were instituted in the Netherlands). We recruited participants through a Dutch commercial research panel, PanelInzicht. Panel members were invited to participate in one of the three online surveys at random. In line with Ton et al. (2022)), we pre‐selected participants with mixed feelings on the debate in question. We were explicit about our interest to participants; the recruitment text asked panel members to participate in the study if they had ‘mixed feelings, doubts, or [had] no clear‐cut opinion about [debate]’, and our informed consent mentioned we were ‘looking for people that had doubts or mixed feelings about [debate]’. Finally, we selected ambivalent individuals via a one‐item self‐report measure, ‘I have doubts or mixed feelings about [debate]’, on a seven‐point Likert scale ranging from 1 = ‘No mixed feelings or doubts at all’ to 7 = ‘Many mixed feelings or doubts.’ The survey was terminated prematurely for participants that reported no doubts or mixed feelings (scores of ‘1’ or ‘2’).

The study was pre‐registered on OSF: https://osf.io/j3rx7. The pre‐registration included the study design and translated items, pre‐planned stopping rule, exclusion criteria and hypothesis. Note that the specific label ‘social discrepancy hypothesis’ was coined after the pre‐registration for communicative purposes. We deviated from the pre‐registered analysis plan by performing exploratory factor analysis and multiple linear regression (with mean scores) instead of conducting structural equation modelling (SEM). We had at least two reasons for this. First, we realized that our final model would result in an SEM model with more degrees of freedom (e.g. df = 195) than the number of participants in our sample (between 181 and 187), which would require us to use mean scores instead of latent factors in the analysis. Second, given that most measures were developed for this study, exploratory factor analysis (in combination with regressions) in hindsight seemed more fitting.

### Participants

We aimed for 200 participants for each societal debate.^1^ We stopped recruiting new participants after 220 completed responses. Note that we did collect more responses in most samples because they started the survey before 220 participants completed the survey. The survey included one attention check.^2^ Participants who failed or did not complete this attention check were removed from the analysis. Detailed information concerning sample exclusion is provided in Table 1.

**TABLE 1:** Sample selection

	Zwarte Piet	Fireworks	Meat consumption
Recruited (accessed survey link)	466	352	339
Excluded observations:			
No consent	113	53	83
Error with data entry^a^	1	1	
Cross‐survey participation^b^	3	6	5
No response after consent	4	4	1
No mixed feelings or doubts	112	45	22
Failed attention check	49	62	41
**Final sample for analysis**	184	181	187

^a^ Unexpectedly, some participants were able to participate in (and complete) multiple surveys. Their responses were removed from all surveys to ensure the independence of the samples.

^b^ In two surveys (Zwarte Piet and Fireworks), one participant (ID) responded to the consent form twice. We assume a technical error because they only completed the survey once. We removed the uncompleted data entry.

#### *Zwarte Piet* debate

In total, 353 panel members gave their consent to participate in this survey. Of these, 233 participants reported mixed feelings or doubts about *Zwarte Piet*. Forty‐nine participants failed the attention check and were excluded. The final sample included 184 participants (*M*_age_ = 48.83, *SD*_age_ = 16.10; 102 female (55%), 80 male (43%) and 2 did not respond). Given the nature of the debate (i.e. about racism), we included a question about participants' self‐reported skin tone. The majority of participants reported a light skin tone (90% reported ‘1’ or ‘2’ on the six‐point self‐report picture measure; Daniel et al., 2009; Fitzpatrick, 1988). In addition, the majority of participants reported celebrating *Sinterklaas* as a child (95%) and having celebrated it in the past 2 years (63%).

#### Fireworks debate

In total, 299 panel members gave their consent to participate in this survey. Of these, 243 participants reported mixed feelings or doubts about *Consumer Fireworks*. Sixty‐two participants failed the attention check. The final sample included 181 participants (*M*_age_ = 52.79, *SD*_age_ = 17.75; 85 (46%) female, 92 (51%) male, 1 other and 3 did not respond). Most participants used to light fireworks on New Year's Eve as a child (53%), but only a minority reported lighting fireworks in the past 2 years (21%).

#### Meat consumption debate

In total, 256 panel members gave their consent to participate in this survey. In total, 228 reported mixed feelings or doubts about *Meat consumption*. Forty‐one participants failed the attention check. The final sample included 187 participants (*M*_age_ = 49.10, *SD*_age_ = 18.07; 101 (54%) female, 82 (44%) male, 2 other and 2 did not respond). The majority of participants ate meat as a child (98%) and in the past month (94%).

### Materials

Table 2 feature the correlation matrices and descriptive statistics of the main variables. Appendix S1 lists items and factor loadings of exploratory factor analysis. Materials, data (excluding exploratory variables and demographic and personal determinants) and code are available in OSF: https://osf.io/rjeh8/.

**TABLE 2:** Correlation matrix and descriptives of the main variables (a) Zwarte Piet; (b) fireworks; (c) meat consumption

		Ambivalence	Discrepancy				
			Personal	Social	Societal		
					Society	Opponents	Proponents
**(a) Zwarte Piet**							
	Mean (*SD*)	2.89 (1.27)	3.42 (1.66)	2.88 (1.32)	3.97 (1.40)	4.43 (1.68)	3.07 (1.60)
Personal	Pearson's *r*	.47***					
	95% CI	[0.35, 0.58]					
Social	Pearson's *r*	.49***	.44***				
	95% CI	[0.20, 0.46]	[0.31, 0.55]				
Societal ‐ Society	Pearson's *r*	.34***	25***	.49***			
	95% CI	[0.20, 0.46]	[0.11, 0.38]	[0.37, 0.59]			
Societal ‐ Opponents	Pearson's *r*	.01	.04	.09	.36***		
	95% CI	[−0.14, 0.15]	[−0.11, 0.18]	[−0.06, 0.23]	[0.23, 0.48]		
Societal ‐ Proponents	Pearson's *r*	.52***	.45***	.67***	.40***	−.03	
	95% CI	[0.40, 0.62]	[0.32, 0.56]	[0.58, 0.74]	[0.27, 0.51]	[−0.18, 0.11]	
**(b) Fireworks**							
	Mean (*SD*)	2.77 (1.12)	4.27 (1.67)	3.20 (1.21)	3.95 (1.32)	3.13 (1.60)	3.93 (1.46)
Personal	Pearson's *r*	.38***					
	95% CI	[0.24, 0.50]					
Social	Pearson's *r*	.44***	.28***				
	95% CI	[0.31, 0.55]	[0.14, 0.41]				
Societal ‐ Society	Pearson's *r*	.02	.07	.32***			
	95% CI	[−0.13, 0.17]	[−0.07, 0.22]	[0.19, 0.45]			
Societal ‐ Opponents	Pearson's *r*	.22**	.01	.27***	.25***		
	95% CI	[0.07. 0.35]	[−0.14, 0.16]	[0.13, 0.40]	[0.11, 0.38]		
Societal ‐ Proponents	Pearson's *r*	.06	.00	.25***	.15*	−.14	
	95% CI	[−0.09, 0.21]	[−0.14, 0.15]	[0.11, 0.38]	[0.01, 0.29]	[−0.28, 0.01]	
**(c) Meat consumption**							
	Mean (*SD*)	3.61 (1.15)	4.42 (1.23)	3.49 (1.17)	3.99 (1.14)	4.18 (1.44)	3.95 (1.35)
Personal	Pearson's *r*	.54***					
	95% CI	[0.43, 0.63]					
Social	Pearson's *r*	.25***	.18*				
	95% CI	[0.11, 0.38]	[0.03, 0.31]				
Societal ‐ Society	Pearson's *r*	.15*	.04	.55***			
	95% CI	[0.00, 0.28]	[−0.10, 0.18]	[0.44, 0.64]			
Societal ‐ Opponents	Pearson's *r*	.01	−.01	.01	.19**		
	95% CI	[0.07, 0.35]	[−0.16, 0.13]	[−0.13, 0.15]	[0.05, 0.33]		
Societal ‐ Proponents	Pearson's *r*	.15*	.17*	.53***	.15*	−.06	
	95% CI	[0.01, 0.29]	[0.03, 0.31]	[0.42, 0.63]	[0.01, 0.29]	[−0.122, 0.08]	

*p < .05, **p < .01, ***p < .001.

### Predictors

#### Social discrepancy

We operationalized social discrepancy as the extent to which people perceive differences in opinion within a particular social group and between the personal opinion and the opinion of a particular social group. Moving beyond previous work, we distinguished between close s*ocial* groups (family, friends, groups they belong to) and more distant *societal* groups (society in general, proponents and opponents of the debate). All items were measured on a seven‐point Likert scale (from 1 = ‘*not at all*’ to 7 = ‘*very much*’).

##### Opinion difference in social groups

We used two items per social group (*family*, *friends and groups they belong to)*. For each group, we measured two types of opinion differences: First, perceived opinion differences between the participants' personal opinion and the opinion of (parts) of their social group were assessed with one item: ‘To what extent do you think that your opinion about [debate] deviates from that of (parts of) [*your family*]?’ Second, perceived opinion differences within one's social group were assessed with a second item: ‘To what extent does the opinion of part of *your family* about [debate] deviate from others in [*your family*]?’. Next to these six items, we added one additional item measuring differences between the opinions of family and their friends: ‘To what extent does the opinion of part of [*your family*] about [debate] deviate from (some of) [*your friends*]?’.

Exploratory factor analysis indicated that all seven items could be combined into one scale with strong internal consistency (Cronbach's alpha: *Zwarte Piet: α* = .93, *Fireworks: α* = .92, *Meat consumption: α* = .90; see Appendix S1, Table A1). Tucker's congruence analysis indicates good similarity [*ϕ* = 1], suggesting that the three factors can be considered equal across topics (see, Lorenzo‐Seva & Berge, 2006). We used a mean score of *perceived social opinion differences* in the analysis.

##### Opinion differences in societal groups

We measured perceived differences in opinion regarding three (more distant) societal groups: *society in general*, *opponents* and *proponents* of [debate]. Again, we measured two types of opinion differences for each group: Firstly, differences between individuals' personal opinions and the opinion of the societal group (‘To what extent do you think that your opinion deviates from that of [*society*] about [debate]?’). Secondly, differences within the societal group (‘To what extent does the opinion of part of [*society*] deviate from others in [*society*] about [debate]?’).

Exploratory factor analysis indicated that these six items formed three meaningful factors, each relating to one of the indicators. The items within these three factors were moderate to strongly correlated (ranging from .38 to .78; see Appendix S1, Table A2). Tucker's congruence analysis indicated fair to good similarity across the three topics: *society* [*ϕ* = .76–.95], *opponents* [*ϕ* = .96–.97], *proponents* [*ϕ* = .83–.88]. For the analysis, we calculated a mean score for each societal group.

#### Personal opinion discrepancy

We measured the discrepancy in participants' personal opinions with one self‐report item that closely resembled the wording of the social discrepancy items: ‘Do you personally have multiple (contradicting) beliefs about [*debate*]?’. This allowed for a methodologically fair comparison with respect to explaining felt ambivalence, as we wanted to test whether perceiving social discrepancy explained ambivalence above and beyond personal opinion discrepancy (see *Analytic Strategy* below).

#### Additional variables

Our survey also included exploratory items (see pre‐registration) that were not included in the analysis of this paper. A short description of these measures and descriptive statistics can be found in Appendix S3.

##### Deviation from pre‐registration

All (social and personal) discrepancy items included the follow‐up question: ‘to what extent is this a problem for you?’. We anticipated that problematic opinion differences would be most strongly related to felt ambivalence. We planned to combine the score on this follow‐up question with the preceding ‘perceived differences’ question, as indicated in the pre‐registration. However, the median, mode and mean for these ‘problem’ items were consistently low, suggesting a floor effect (see Table B1 in Appendix S2). We interpreted this floor effect as an indication that participants did not experience the discrepancy as a ‘problem’. This suggested that the discrepancy items were more relevant than the problem items. Therefore, we decided to exclude the ‘problem’ items from the analysis.^3^ We should note, however, that it is possible that the wording of this item was too extreme: Participants might still have experienced discomfort, which we, unfortunately, did not ask.

### Dependent variable

#### Felt ambivalence

Felt ambivalence about the debate was assessed with eight items adapted from the Melbourne decision‐making questionnaire (Mann et al., 1997) and the three‐item measure of subjective ambivalence (Priester & Petty, 1996). For example, ‘If I do not have to, I prefer not taking a position about X,’ ‘I feel conflicted about X.’ Scale endpoints ranged from 1 = ‘Completely disagree’ to 7 = ‘Completely agree’. Exploratory factor analysis confirmed that the scale could not be divided into meaningful subsets and had good internal consistency (Cronbach's alpha: *Zwarte Piet*, *α* = .87; *Fireworks*, *α* = .82; *Meat consumption*, *α* = .85; see Appendix S1, Table A3). Additionally, Tucker's congruence analysis indicates good similarity [*ϕ* = 0.97–0.99], suggesting that the three factors can be considered equal across topics (Lorenzo‐Seva & Berge, 2006). We used a mean score of the eight items in our analysis.

### Analytic strategy

All analyses reported in this manuscript were conducted in R (version 4.0.3; R Core Team, 2020). For the analysis, we used the R packages ‘stats’ (R Core Team, 2020), ‘psych’ (version 2.0.8; Revelle, 2020), ‘car’ (Fox & Weisberg, 2019) and ‘effectsize’ (Ben‐Shachar et al., 2020). Materials, data and code are available in OSF. To test the *social discrepancy hypothesis*, we conducted multiple linear regression (MLR) with felt ambivalence as the dependent measure. Predictors were added in three steps (comparable to a hierarchical MLR with the ‘enter method’ in SPSS). We were interested in the *R* ^2^‐increase since this could indicate the added value of the social discrepancy indicators (additional explained variance). Therefore, Step 1 tested the base hypothesis that personal opinion discrepancy relates to felt ambivalence. Step 2a and Step 2b allowed us to test the *social discrepancy hypothesis* by calculating the added value of perceived opinion differences in social groups (2a) and perceived opinion differences in societal groups (2b) above and beyond personal opinion discrepancy. Finally, Step 3 allowed us to compare and gave us insight into the relevance of each of the predictors.

#### Power

Given a sample size of 180 participants using sensitivity analysis in G*Power (Faul et al., 2009), we should be able to detect an *R* ^2^‐increase with an effect size (f^2^) of 0.06 or more with 80% power in a multiple linear regression with three tested predictors and a total of four predictors (i.e., Step 2b, see Figure 1 in Appendix S1).

## RESULTS

Table 3 report the inferential statistics of the multiple regressions. We report the findings below, organized by issue.

**TABLE 3:** Multiple linear regression predicting felt ambivalence concerning (a) Zwarte Piet; (b) Fireworks^a^; (c) Meat consumption

Step		Unstandardized					*η* ^*2*^ *p*	$R_{\text{adj}}^{2}$.	$R_{\text{change}}^{2}$	*F*	*df*	*p*
		*B*	*SE*	*p*	*β*	95% CI						
**(a) Zwarte Piet**												
1	Personal	0.36	0.05	<.01	.47	[0.34, 0.60]	.22	.22		51.47	1, 182	<.01
2a	Personal	0.24	0.05	<.01	.32	[0.18, 0.45]	.25	.32	.10	42.98	2, 181	<.01
	Social	0.34	0.07	<.01	.35	[0.22, 0.49]	.13					
2b	Personal	0.22	0.05	<.01	.29	[0.15, 0.42]	.26	.34	.12	24.50	4, 178	<.01
	Societal ‐ Society	0.14	0.07	.03	.15	[0.01, 0.30]	.07					
	Societal ‐ Opponents	−0.04	0.05	.47	−.05	[−0.18, 0.08]	.02					
	Societal ‐ Proponents	0.26	0.06	<.01	.32	[0.18, 0.47]	.10					
3	Personal	0.20	0.05	<.01	.26	[0.13, 0.40]	.26	.35		20.64	5, 177	<.01
	Social	0.16	0.08	.06	.17	[0.00, 0.34]	.14					
	Societal ‐ Society	0.10	0.07	.13	.11	[−0.03, 0.26]	.02					
	Societal ‐ Opponents	−0.04	0.05	.45	−.05	[−0.18, 0.08]	.01					
	Societal ‐ Proponents	0.19	0.07	<.01	.24	[0.07, 0.41]	.04					
**(b) Fireworks**												
1	Personal	0.26	0.05	<.01	.38	[0.24, 0.51]	.14	.14		29.51	1, 178	<.01
2a	Personal	0.19	0.05	<.01	.27	[0.14, 0.41]	.16	.25	.12	31.31	2, 177	<.01
	Social	0.33	0.06	<.01	.36	[0.23, 0.49]	.14					
2b	Personal	0.25	0.05	<.01	.37	[0.24, 0.51]	.15	.18	.04	10.76	4, 175	<.0.1
	Societal ‐ Society	−0.07	0.06	.24	−.08	[−0.23, 0.06]	.00					
	Societal ‐ Opponents	0.17	0.05	<.01	.24	[0.10, 0.06]	.06					
	Societal ‐ Proponents	0.08	0.05	.14	.10	[−0.03, 0.24]	.00					
3	Personal	0.19	0.05	<.01	.28	[0.15, 0.41]	.17	.28		14.66	5, 174	<.01
	Social	0.34	0.07	<.01	.37	[0.22, 0.51]	.15					
	Societal ‐ Society	−0.14	0.06	.02	−.16	[−0.30, −0.02]	.02					
	Societal ‐ Opponents	0.11	0.05	.03	.15	[0.01, 0.29]	.03					
	Societal ‐ Proponents	0.01	0.05	.88	.01	[−0.12, 0.15]	.00					
**(c) Meat consumption**												
1	Personal	0.51	.06	<.01	.54	[0.42, 0.66]	.29	.29		75.62	1, 185	<.01
2a	Personal	0.48	.06	<.01	.51	[0.39, 0.63]	.30	.31	.02	42.19	2, 184	<.01
	Social	0.16	.06	.01	.16	[0.04, 0.28]	.03					
2b	Personal	0.50	0.06	<.01	.53	[0.41,0.66]	.29	.29	.004	20.02	4, 182	<.01
	Societal ‐ Society	0.12	0.07	.08	.12	[−0.02, 0.26]	.02					
	Societal ‐ Opponents	−0.002	0.05	.97	0	[−0.13, 0.12]	.00					
	Societal ‐ Proponents	0.005	0.06	.94	0	[−0.13, 0.15]	.00					
3	Personal	0.49	.06	<.01	.52	[0.39, 0.64]	.30	.30		16.86	5, 181	<.01
	Social	0.14	.08	.07	.14	[−0.01, 0.30]	.03					
	Societal ‐ Society	0.06	.08	.42	.06	[−0.09, 0.22]	.00					
	Societal ‐ Opponents	0.003	.05	.95	.0001	[−0.12, 0.13]	.00					
	Societal ‐ Proponents	−0.04	.06	.57	−.04	[−0.19, 0.11]	.00					

*Note*: $R_{\text{change}}^{2}$ was calculated with respect to Step 1.

^a^ In this sample, one observation was excluded due to missingness.

### Zwarte Piet debate

*Step 1: Personal Opinion Discrepancy*. In line with the broader literature, personal opinion discrepancy was associated with felt ambivalence and explained 22% of the variance in felt ambivalence, *F* (1, 181) = 51.47, *p* < .01.
*Step 2: Social Discrepancy*.
*Step 2a: Opinion Differences in Social Groups*. In line with our *social discrepancy hypothesis*, perceiving opinion differences in social groups together with personal opinion discrepancy explained felt ambivalence, *F* (2, 180) = 42.98, *p* < .01. More so, the explained variance increased to 32%, indicating that opinion differences in social groups explained 10% additional variance in felt ambivalence.
*Step 2b: Opinion Differences in Societal Groups*. Similarly, in line with our *social discrepancy hypothesis*, perceiving opinion differences in societal groups together with personal opinion discrepancy explained felt ambivalence, *F* (4, 178) = 24.50, *p* < .01. Indeed, the explained variance increased to 34%, suggesting that opinion differences in societal groups, specifically regarding differences in *proponents* of Zwarte Piet (*β* = .26) and in *society* (*β* = .15), explained 12% additional variance.
*Step 3: Final Model*. The final model indicated a positive association between the predictors and felt ambivalence, *F* (5, 177) = 20.64, *p* < .01, explaining 35% of the variance in felt ambivalence. Interestingly, above and beyond personal discrepancy (*β* = .26), perceiving differences in *proponents* (*β* = .19) was a significant predictor. Opinion differences in social groups (*β* = .17), perceiving differences in *society* and *opponents* were not significant (*β* = .11; *β* = −.05). This indicates that, for this topic, perceiving differences in proponents of *Zwarte Piet* was a better predictor for felt ambivalence than perceiving differences in social groups.

### Fireworks debate

*Step 1: Personal Opinion Discrepancy*. In line with our previous findings, personal opinion discrepancy was associated with felt ambivalence, *F* (1, 178) = 29.51, *p* < .01 and explained 14% of the variance in felt ambivalence.
*Step 2: Social Discrepancy*
*Step 2a: Opinion Differences in Social Groups*. Supporting the *social discrepancy hypothesis*, perceiving opinion differences in social groups and personal opinion discrepancy explained felt ambivalence, *F* (2, 177) = 31.31, *p* < .01. The explained variance improved to 25%, suggesting that opinion differences in social groups explained 11% additional variance in felt ambivalence.
*Step 2b: Opinion Differences in Societal Groups*. In support of the *societal discrepancy hypothesis*, perceiving opinion differences in societal groups and personal opinion discrepancy explained felt ambivalence, *F* (4, 175) = 10.76, *p* < .01. Again, the explained variance improved to 18%, suggesting that opinion differences in societal groups, specifically in *opponents* of Fireworks (*β* = .24), explained 4% additional variance.
*Step 3: Final Model*. The final model indicated a positive association between the predictors and felt ambivalence, *F*(5, 174) = 14.66, *p* < .01, which explained 28% of the variance in felt ambivalence. Opinion differences in social groups (*β* = .37) were the most relevant predictor, together with personal discrepancy (*β* = .28) and perceived differences in *opponents of Fireworks* (*β* = .15). Perceiving differences in *proponents of Fireworks* (*β* = .01) was not significant, whereas, somewhat surprisingly, perceiving differences in *society* (*β* = −.15) was negatively associated with felt ambivalence.

### Meat consumption debate

*Step 1: Personal Opinion Discrepancy*. In line with our previous findings, personal opinion discrepancy was positively associated with felt ambivalence, *F* (1, 185) = 75.62, *p* < .01 and explained 29% of the variance in felt ambivalence.
*Step 2: Social Discrepancy*
*Step 2a: Opinion Differences in Social Groups*. Perceiving *opinion differences in social groups* and personal opinion discrepancy explained *F* (2, 184) = 42.19, *p* < .01, with an overall explained variance of 31%. In support of the *social discrepancy hypothesis*, opinion differences in social groups explained a 2% additional variance of felt ambivalence. This percentage was considerably smaller than for the other two topics.
*Step 2b: Opinion Differences in Societal Groups*. Although the overall model was significant, *F* (4, 182) = 20.02, *p* < .01, in contrast to the previous findings, the explained variance did not improve compared to Step 1.
*Step 3: Final Model*. The regression equation of the full model was significant, *F* (5, 181) = 16.86, *p* < .01 and explained 30% of the variance in felt ambivalence. However, for this topic, only personal opinion discrepancy (*β* = .52) was a significant predictor for felt ambivalence in the context of meat consumption. Indeed, counter the *social discrepancy hypothesis*, the effects of perceived opinion differences in *social groups* (*β* = .14) and differences in *society*, *opponents and proponents* (*β* = .06; *β* = .0001; *β* = −.04) were not significant.

## DISCUSSION

The current work put forth and tested the *social discrepancy hypothesis*: The idea that, among ambivalents, perceived discrepancies in opinions about the debate in both social (e.g. friends, family, social) and societal (e.g. society, proponents, opponents) groups are positively associated with the experience of ambivalence. This hypothesis was tested quantitatively in the context of three different societal debates in the Netherlands (*Zwarte Piet*, New Year's Eve fireworks, meat consumption). In line with the social discrepancy hypothesis, we found that both perceived opinion differences in social and societal groups explained felt ambivalence above and beyond personal opinion discrepancy. Indeed, depending on the debate, social discrepancy improved the explained variance of the model by up to 12%. These findings confirm that social discrepancy can be associated with the experience of ambivalence in the context of societal debates, reflecting the social tensions felt within.

Importantly, we were able to generalize this finding across different societal debates and differentiate between social and societal forces. Whereas most quantitative work on felt ambivalence has focused on how discrepancies in *personal* beliefs and opinions affect experiences of ambivalence (i.e. having mixed reactions, feeling torn and conflicted and/or being undecided about an issue; Priester & Petty, 1996; van Harreveld, Rutjens, et al., 2009; van Harreveld, van der Pligt, et al., 2009), we found that in the context of polarized societal debates, other more socially oriented forces, such as opinion differences of friends, family or societal groups, come into play. This moves our work beyond previous work on interpersonal discrepancy (Cowley & Czellar, 2012; Priester & Petty, 2001) to also include perceived opinion differences in social and societal groups that come into play in polarized contexts. Importantly, our quantitative findings corroborate the qualitative finding that people felt ambivalent because they found themselves caught in the social crossfire of opposing positions held by social forces (a mix of social and societal groups) in the context of the *Zwarte Piet* debate (Ton et al., 2022). We move beyond these findings by differentiating multiple social discrepancy predictors and testing the social discrepancy hypothesis across multiple polarized societal debates.

Intriguingly, we observed contextual variation in the predictive value of the different discrepancy predictors. In the context of *Zwarte Piet*, perceiving differences among societal groups, specifically proponents of *Zwarte Piet*, and in the context of fireworks, opinion differences in social groups (family, friends and other groups) seemed to be the most relevant social discrepancy predictors. These findings suggest that, for ambivalents, the debate about fireworks has a more substantial interpersonal or relational component, and the *Zwarte Piet* debate focuses more on societal groups. Here, individuals would need to reconcile the different opinions of these groups and their personal opinion discrepancy to overcome their ambivalence. More so, in the fireworks debate, the social component was just as, if not more, predictive of felt ambivalence as personal opinion differences. Interestingly, and by contrast, for meat consumption, most variance in felt ambivalence could be explained by personal opinion discrepancy, suggesting that the social tensions were not as relevant in this particular debate. Below we discuss the implications and limitations of our findings with an eye to future research.

### Implications

Our findings imply, first and foremost, that theory and research on ambivalence should include a stronger focus on the social tensions felt among ambivalents in the context of polarized societal debates. Indeed, for the *Zwarte Piet* and firework debates at least, our findings suggest that, among ambivalents, perceiving differences in society and the direct social environment are positively related to feelings of ambivalence. This implies that the opinion differences people will perceive in polarized debates are likely to contribute to their experience of ambivalence (see also Keele & Wolak, 2008). While polarized debates may breed further polarization for those who take sides (e.g. Dunlap et al., 2016; Wilson et al., 2020), it can paradoxically induce or strengthen ambivalence for those with mixed opinions. This implies for theory and research on social change that polarized debates may both strengthen and weaken chances of social change. More generally, our work emphasizes the importance of social forces in understanding how people navigate societal debates (Agostini & van Zomeren, 2021; Dovidio et al., 2009; Jost et al., 2017) and of individuals' psychological embeddedness in interpersonal networks, psychological groups and broader social systems (van Zomeren, 2016).

One potential explanation for the mixed findings is that the debate on meat consumption may be perceived to be less polarized than the other two debates. Although we cannot empirically verify this in the current study, the debate on meat consumption is not as binary as the other debates (one can take multiple possible positions, including vegan, vegetarian, flexitarian, reduced consumption, conscious [bio] consumption, carnivore; see also Thomas et al., 2019). This abundance of groups might dilute the divide and distinction between the two opinion‐based groups characterizing polarized debates, making the debate concerning meat consumption feel less polarized. If true, our findings would be consistent with the idea that social discrepancy is particularly important in *polarized* societal debates. Future research can examine this in more detail.

The divergent finding for the meat consumption debate is also interesting as it indicates that social discrepancy does not contribute to ambivalence in all societal debates, which necessitates a closer analysis of the kind of debates in which social discrepancy may (not) play a role. One apparent difference between the meat consumption debate and the other two debates is that, in the meat consumption debate, a more personal behavioural (consumer and dietary) decision is salient, which requires individuals to (re)consider their habits and attitudes towards meat (see, Pauer et al., 2022; Rothgerber, 2014). Participants' responses to an exploratory rank‐order question confirm the personal relevance of this context (see Appendix S3, Table C2); the majority (67%) of participants chose ‘self’ as a reason for ambivalence, compared with 26% in the *Zwarte Piet* debate and 41% in the fireworks debate. A potential explanation could be that perceiving social discrepancy mainly plays a role for felt ambivalence about social topics that imply social or societal action. It might, thus, be relevant to consider whether the proposed social change entails personal versus social or societal action. We should note we did not study the link between ambivalence and action in this research. Other work suggests that, generally, ambivalence makes people avoid decisions. We wonder whether the same can be predicted for ambivalents in the context of polarized social debates, as felt ambivalence may also indicate a potential acceptance of change in a particular direction. Future research can explore this interesting direction.

We want to emphasize that our findings do *not* imply that (intra)personal discrepancy is unimportant for ambivalence in polarized social contexts. In fact, personal opinion discrepancy was a consistent predictor of ambivalents' felt ambivalence in and across the three debates. As such, our findings do not contradict the assumption in current ambivalence research that intrapersonal discrepancy causes feelings of ambivalence (e.g., Jonas et al., 2000; Newby‐Clark et al., 2002; Schneider & Schwarz, 2017; Thompson et al., 1995). Nevertheless, our perspective emphasizes the importance of integrating the social tensions that come with polarized societal debates into the experience of ambivalence. Given this focus on feeling caught in a social crossfire (Ton et al., 2022), our research offers specific predictors of the experience of ambivalence that move beyond cognitive discrepancy (adding to predictors proposed in other lines of research, see DeMarree et al., 2014; Keele & Wolak, 2008; Priester & Petty, 2001). To foster further connections, future research could refine^4^ the measures developed for this study and compare them with other more established measures in the ambivalence literature (e.g. personal opinion discrepancy versus objective ambivalence; Thompson et al., 1995).

### Limitations and future directions

Future research can also help solve limitations of the current research. For instance, research can target ambivalents and polarized debates on different topics and in different countries, as this will help establish further evidence for the external validity of the social discrepancy hypothesis. Furthermore, given that our findings are cross‐sectional, they do not offer evidence for the internal validity of the causal direction implied in the social discrepancy hypothesis. Experimental methods can be used to resolve this. Indeed, it might be possible that opinion differences are more salient for ambivalent individuals and that, subsequently, the salience of these opinion differences could make people feel more ambivalent. Nevertheless, we assume that in polarized contexts, these opinion differences are clearly (and often) communicated in society through (social) media and discussions with those in one's network, and hence are easily perceived to influence how ambivalents feel. Future longitudinal research, in which ambivalent individuals are followed while a societal debate develops (e.g. in anticipation of a referendum or policy change), may provide key insights into the causal relationship between social discrepancy and felt ambivalence over time.

Furthermore, this work generates exciting questions for future research about the need to understand the role of perceived polarization (e.g. Koudenburg & Kashima, 2021). Future research should take a closer look into whether, for ambivalents, more polarized debates offer a stronger breeding ground for ambivalence. For example, future research could compare social discrepancy and ambivalence in different countries where the same debate is perceived as more or less polarized. In addition, including measures of perceived polarization in future research will also help to test our suggestion that polarization can breed ambivalence. Moreover, research could compare ambivalents with non‐ambivalents in the same societal debates to test whether the relation between perceived social discrepancy and felt ambivalence is unique to ambivalents.

In conclusion, the findings of this research corroborate and move beyond previous work by affirmatively answering the question of whether, among ambivalents, the social tensions of polarized societal debates are felt within. The findings also raise new theoretical and empirical questions about ambivalence in polarized societal debates with an eye to social change and provide directions for future research. Specifically, by providing quantitative evidence for the *social discrepancy hypothesis*, we believe that theory and research should pay more attention to ambivalents in the context of polarized societal debates. As they feel caught in the social crossfire of such debates, their felt ambivalence is of a highly social nature, reflecting the social tensions felt within.

## AUTHOR CONTRIBUTIONS

**Gonneke Marina Ton:** Conceptualization; data curation; investigation; methodology; writing – original draft; writing – review and editing. **Katherine Stroebe:** Conceptualization; methodology; supervision; writing – original draft; writing – review and editing. **Martijn van Zomeren:** Conceptualization; methodology; supervision; writing – original draft; writing – review and editing.

## CONFLICT OF INTEREST

None.

### OPEN RESEARCH BADGES

This article has earned Open Data, Open Materials and Preregistered Research Design badges. Data, materials and the preregistered design and analysis plan are available at https://osf.io/rjeh8/.

## Supporting information


Appendix S1–S3

## Data Availability

The data that support the findings are archived and available in OSF: https://osf.io/rjeh8/.

## Footnotes

^1^
We initially planned to use Structural Equation Modelling (SEM) in this study; Monte Carlo Simulation studies on SEM indicated that, in general, larger samples (e.g. 200 or more) yielded more precision and better estimations (Loehlin et al., 2016). Based on financial considerations, we decided to aim for 200 participants per sample.

^2^
We included one attention check in between the items of Felt Ambivalence (DV). Participants were asked to select the option ‘1. Completely Disagree’.

^3^
In addition, exploratory factor analyses indicated that the ‘problem’ versus ‘perceived differences’ items loaded on different factors across all three data sets (see Table B2 in Appendix S2). Furthermore, including these as separate predictors in multiple regression analyses brought a high degree of multicollinearity (for VIF‐values of Multiple Regression including these items, see Table B3 in Appendix S2).

^4^
As noted on p. 13, we anticipated that opinion differences that were perceived to be problematic would be most strongly related to felt ambivalence. However, participants did not experience a “problem”. Nonetheless, participants might still have experienced discomfort, which could be incorporated in future research.

## References

[bjso12574-bib-0001] Agostini, M. , & van Zomeren, M. (2021). Toward a comprehensive and potentially cross‐cultural model of why people engage in collective action: A quantitative research synthesis of four motivations and structural constraints. Psychological Bulletin, 147(7), 667–700. 10.1037/bul0000256 34855427

[bjso12574-bib-0003] Ben‐Shachar, M. S. , Lüdecke, D. , & Makowski, D. (2020). Effectsize: Estimation of effect size indices and standardized parameters. Journal of Open Source Software, 5(56), 2815. 10.21105/joss.02815

[bjso12574-bib-0004] Bliuc, A.‐M. , Bouguettaya, A. , & Felise, K. D. (2021). Online intergroup polarization across political fault lines: An integrative review. Frontiers in Psychology, 12, 641215. 10.3389/fpsyg.2021.641215 34733195 PMC8559783

[bjso12574-bib-0005] Bliuc, A.‐M. , McGarty, C. , Reynolds, K. , & Muntele, D. (2007). Opinion‐based group membership as a predictor of commitment to political action. European Journal of Social Psychology, 37(1), 19–32. 10.1002/ejsp.334

[bjso12574-bib-0006] Cowley, E. , & Czellar, S. (2012). The moderating role of self‐monitoring on the interpersonal aspects of attitude ambivalence. Journal of Personality, 80(4), 949–968. 10.1111/j.1467-6494.2011.00754.x 22092026

[bjso12574-bib-0007] Daniel, L. C. , Heckman, C. J. , Kloss, J. D. , & Manne, S. L. (2009). Comparing alternative methods of measuring skin color and damage. Cancer Causes & Control, 20(3), 313–321. 10.1007/s10552-008-9245-3 18931926 PMC2702995

[bjso12574-bib-0008] DeMarree, K. G. , Christian Wheeler, S. , Briñol, P. , & Petty, R. E. (2014). Wanting other attitudes: Actual–desired attitude discrepancies predict feelings of ambivalence and ambivalence consequences. Journal of Experimental Social Psychology, 53, 5–18. 10.1016/j.jesp.2014.02.001

[bjso12574-bib-0009] Dovidio, J. F. , Gaertner, S. L. , & Saguy, T. (2009). Commonality and the complexity of “we”: Social attitudes and social change. Personality and Social Psychology Review, 13(1), 3–20. 10.1177/1088868308326751 19144903

[bjso12574-bib-0010] Dunlap, R. E. , McCright, A. M. , & Yarosh, J. H. (2016). The political divide on climate change: Partisan polarization widens in the US. Environment: Science and Policy for Sustainable Development, 58(5), 4–23. 10.1080/00139157.2016.1208995

[bjso12574-bib-0011] Faul, F. , Erdfelder, E. , Buchner, A. , & Lang, A.‐G. (2009). Statistical power analyses using G*power 3.1: Tests for correlation and regression analyses. Behavior Research Methods, 41(4), 1149–1160. 10.3758/BRM.41.4.1149 19897823

[bjso12574-bib-0012] Fitzpatrick, T. B. (1988). The validity and practicality of sun‐reactive skin types I through VI. Archives of Dermatology, 124(6), 869–871. 10.1001/archderm.1988.01670060015008 3377516

[bjso12574-bib-0013] Fox, J. , & Weisberg, S. (2019). An {R} companion to applied regression: Vol (3rd ed.). SAGE.

[bjso12574-bib-0014] Jonas, K. , Broemer, P. , & Diehl, M. (2000). Attitudinal ambivalence. European Review of Social Psychology, 11(1), 35–74. 10.1080/14792779943000125

[bjso12574-bib-0015] Jost, J. T. , Becker, J. , Osborne, D. , & Badaan, V. (2017). Missing in (collective) action: Ideology, system justification, and the motivational antecedents of two types of protest behavior. Current Directions in Psychological Science, 26(2), 99–108. 10.1177/0963721417690633

[bjso12574-bib-0016] Keele, L. , & Wolak, J. (2008). Contextual sources of ambivalence. Political Psychology, 29(5), 653–673. 10.1111/j.1467-9221.2008.00659.x

[bjso12574-bib-0017] Koudenburg, N. , & Kashima, Y. (2021). A polarized discourse: Effects of opinion differentiation and structural differentiation on communication. Personality and Social Psychology Bulletin, 48, 1068–1086. 10.1177/01461672211030816 34292094 PMC9178781

[bjso12574-bib-0018] Loehlin, J. C. , Beaujean, A. A. , & Beaujean, A. A. (2016). Latent variable models: An introduction to factor, path, and structural equation analysis (5th ed.). Routledge. 10.4324/9781315643199

[bjso12574-bib-0019] Lorenzo‐Seva, U. , & Berge, J. (2006). Tucker's congruence coefficient as a meaningful index of factor similarity. Methodology, 2, 57–64. 10.1027/1614-2241.2.2.57

[bjso12574-bib-0020] Mann, L. , Burnett, P. , Radford, M. , & Ford, S. (1997). The Melbourne decision making questionnaire: An instrument for measuring patterns for coping with decisional conflict. Journal of Behavioral Decision Making, 10(1), 1–19. 10.1002/(SICI)1099-0771(199703)10:1<1::AID-BDM242>3.0.CO;2-X

[bjso12574-bib-0021] McGarty, C. , Bliuc, A.‐M. , Thomas, E. F. , & Bongiorno, R. (2009). Collective action as the material expression of opinion‐based group membership. Journal of Social Issues, 65(4), 839–857. 10.1111/j.1540-4560.2009.01627.x

[bjso12574-bib-0022] Newby‐Clark, I. , McGregor, I. , & Zanna, M. (2002). Thinking and caring about cognitive inconsistency: When and for whom does attitudinal ambivalence feel uncomfortable? Journal of Personality and Social Psychology, 82(2), 157–166. 10.1037//0022-3514.82.2.157 11831406

[bjso12574-bib-0023] Pauer, S. , Ruby, M. , Rutjens, B. , Perino, G. , & van Harreveld, F. (2022). Meating conflict: Toward a model of ambivalence‐motivated meat reduction. Foods, 11(7), 921. 10.3390/foods1107092 35407008 PMC9040712

[bjso12574-bib-0024] Pillaud, V. , Cavazza, N. , & Butera, F. (2013). The social value of being ambivalent: Self‐presentational concerns in the expression of attitudinal ambivalence. Personality and Social Psychology Bulletin, 39(9), 1139–1151. 10.1177/0146167213490806 23761925

[bjso12574-bib-0025] Priester, J. R. , & Petty, R. E. (1996). The gradual threshold model of ambivalence: Relating the positive and negative bases of attitudes to subjective ambivalence. Journal of Personality and Social Psychology, 71(3), 431–449. 10.1037/0022-3514.71.3.431 8831157

[bjso12574-bib-0026] Priester, J. R. , & Petty, R. E. (2001). Extending the bases of subjective attitudinal ambivalence: Interpersonal and intrapersonal antecedents of evaluative tension. Journal of Personality and Social Psychology, 80(1), 19–34. 10.1037//0022-3514.80.1.19 11195888

[bjso12574-bib-0027] R Core Team . (2020). R: A language and environment for statistical computing (version 4.0.3) [computer software]. R Foundation for Statistical Computing. https://www.R‐project.org/

[bjso12574-bib-0028] Revelle, W. (2020). Psych: Procedures for psychological, psychometric, and personality research (version 2.0.8) [computer software]. Northwestern University. https://CRAN.R‐project.org/package=psych

[bjso12574-bib-0029] Rosenfeld, D. L. (2018). The psychology of vegetarianism: Recent advances and future directions. Appetite, 131, 125–138. 10.1016/j.appet.2018.09.011 30227184

[bjso12574-bib-0030] Rothgerber, H. (2014). Efforts to overcome vegetarian‐induced dissonance among meat eaters. Appetite, 79, 32–41. 10.1016/j.appet.2014.04.003 24727102

[bjso12574-bib-0031] Ruby, M. B. (2012). Vegetarianism. A blossoming field of study. Appetite, 58(1), 141–150. 10.1016/j.appet.2011.09.019 22001025

[bjso12574-bib-0032] Schneider, I. K. , & Schwarz, N. (2017). Mixed feelings: The case of ambivalence. Current Opinion in Behavioral Sciences, 15, 39–45. 10.1016/j.cobeha.2017.05.012

[bjso12574-bib-0033] Thomas, E. F. , Bury, S. M. , Louis, W. R. , Amiot, C. E. , Molenberghs, P. , Crane, M. F. , & Decety, J. (2019). Vegetarian, vegan, activist, radical: Using latent profile analysis to examine different forms of support for animal welfare. Group Processes & Intergroup Relations, 22(6), 836–857. 10.1177/1368430218824407

[bjso12574-bib-0034] Thompson, M. M. , Zanna, M. P. , & Griffin, D. W. (1995). Let's not be indifferent about (attitudinal) ambivalence. In R. E. Petty & J. A. Krosnick (Eds.), Attitude strength: Antecedents and consequences (pp. 361–386). Lawrence Erlbaum Associates, Inc.

[bjso12574-bib-0035] Ton, G. M. , Stroebe, K. , & van Zomeren, M. (2022). Caught in a social crossfire: Exploring the social forces behind and experience of ambivalence about potential social change. European Journal of Social Psychology, 52(1), 147–159. 10.1002/ejsp.2821

[bjso12574-bib-0036] van Harreveld, F. , Nohlen, H. U. , & Schneider, I. K. (2015). The ABC of ambivalence: Affective, behavioral, and cognitive consequences of attitudinal conflict. Advances in Experimental Social Psychology, 52, 285–324. 10.1016/bs.aesp.2015.01.002

[bjso12574-bib-0037] van Harreveld, F. , Rutjens, B. T. , Rotteveel, M. , Nordgren, L. F. , & van der Pligt, J. (2009). Ambivalence and decisional conflict as a cause of psychological discomfort: Feeling tense before jumping off the fence. Journal of Experimental Social Psychology, 45(1), 167–173. 10.1016/j.jesp.2008.08.015

[bjso12574-bib-0038] van Harreveld, F. , van der Pligt, J. , & de Liver, Y. N. (2009). The agony of ambivalence and ways to resolve it: Introducing the MAID model. Personality and Social Psychology Review, 13(1), 45–61. 10.1177/1088868308324518 19144904

[bjso12574-bib-0039] van Zomeren, M. (2016). From self to social relationships: An essentially relational perspective on social motivation. Cambridge University Press.

[bjso12574-bib-0040] Wilson, A. E. , Parker, V. A. , & Feinberg, M. (2020). Polarization in the contemporary political and media landscape. Current Opinion in Behavioral Sciences, 34, 223–228. 10.1016/j.cobeha.2020.07.005

