# Supplementary material for: The social tensions felt within: Explaining felt ambivalence about polarized societal debates through perceived opinion discrepancies in the social environment
Source: Br J Soc Psychol. 2022 Sep 11;62(1):30–46. doi: 10.1111/bjso.12574 (PMC10087039; doi:10.1111/bjso.12574)
Supplement: Supplementary file 1 — Appendix S1–S3 [file BJSO-62-30-s001.pdf]

Appendix I

Main Items: Factor Loadings of Exploratory Factor Analysis.

Exploratory factor analysis, Cronbach's alpha, and Tucker's congruence analysis reported in this manuscript were calculated using the R package "psych" (Revelle, 2020). For the exploratory factor analysis, we used the default settings: minimum residual solution with oblimin transformation.

Table A1. Opinion Differences in Social Groups:
Items, Factor Loadings (one-factor solution) and Cronbach's Alpha

	**Zwarte Piet** α = .93	**Fireworks** α = .92	**Meat consumption** α = .90
1. Does *your opinion* deviates from that of (parts of) *your family*	0.83	0.76	0.66
2. Does the opinion of part of *your family* deviate from others in *your family*	0.83	0.76	0.66
3. Does *your opinion* deviates from that of (some of) *your friends*	0.85	0.84	0.78
4. Does the opinion of some of *your friends* deviate from others of *your friends*	0.84	0.83	0.80
5. Does the opinion of (part of) *your family* deviate from (some of) *your friends*	0.87	0.81	0.81
6. Does *your opinion* deviates from that of *groups you belong to*	0.82	0.78	0.74
7. Does the opinion of some of *the groups you belong to* deviate from *other groups you belong to*	0.84	0.79	0.76

Table A2. Opinion Differences in Societal Groups:
Items, Factor Loadings (three-factor solution), and Pearson's Correlation

	Zwarte Piet				Fireworks				Meat consumption			
	F1	F2	F3	*r*	F1	F2	F3	*r*	F1	F2	F3	*r*
1. Does *your opinion* deviates from that of (parts of) *society*	0.00	-0.02	**1.01**	.56	0.07	**0.51**	0.16	.59	0.00	-0.05	**0.91**	.50
2 Does the opinion of part of *society* deviate from others in *society*	0.26	0.23	**0.42**		0.00	**1.00**	-0.03		0.21	0.21	**0.42**	
1. Does *your opinion* deviates from that of (parts of) *opponents of X*	**0.88**	-0.10	-0.01	.65	**1.00**	-0.02	-0.02	.78	**0.99**	0.04	-0.01	.54
2 Does the opinion of *opponents* deviate from other *opponents of X*	**0.77**	0.11	0.05		**0.76**	0.08	0.06		**0.53**	0.26	0.10	
1. Does *your opinion* deviates from that of (parts of) *proponents of X*	-0.15	**0.80**	0.08	.66	-0.33	0.19	0.29	.38	-0.23	**0.31**	**0.39**	.48
2 Does the opinion of *proponents* deviate from other *proponents of X*	0.13	**0.79**	-0.06		0.01	-0.01	**1.00**		-0.01	**1.00**	-0.02	

Note. Factor loadings greater than |0.30| are shown in boldface. Boxes indicate the items we combined into one mean score.

Table A3. Felt Ambivalence:
Items, Factor Loadings (one-factor solution) and Cronbach's Alpha

	**Zwarte Piet** α = .87	**Fireworks** α = .82	**Meat consumption** α = .85
1. I feel conflicted about [*debate*].	0.77	0.73	0.80
2. If I really don't have to, I'd rather not take a position on [...].	0.61	0.59	0.45
3. I am indecisive about [...].	0.83	0.60	0.76
4. I feel torn about [...].	0.44	0.73	0.54
5. I am sure about [...]. (RC)	0.44	0.30	0.51
6. I have mixed feelings about [...].	0.79	0.60	0.78
7. I avoid taking a stand on [...].	0.77	0.68	0.53
8. I have doubts about [...]	0.78	0.67	0.76

Note. The fifth (reversed coded) item had a relatively low factor loading in all three samples. However, there was no consistent, substantial improvement in the Cronbach's alpha after removing the item from the scale: $\alpha_{\text{Zwarte Piet}}$= .88; $\alpha_{\text{Fireworks}}$= .84; $\alpha_{\text{Meat consumption}}$ = .84. Therefore, we decided to retain the full scale.

Figures

Figure 1

Sensitivity Analysis for Multiple Regression, G*Power (Faul et al., 2009), we should be able to detect an R^2-increase with an effect size (f^2) of 0.06 or more with 80% power in a multiple linear regression with three tested predictors and a total of four predictors (Step 2b).

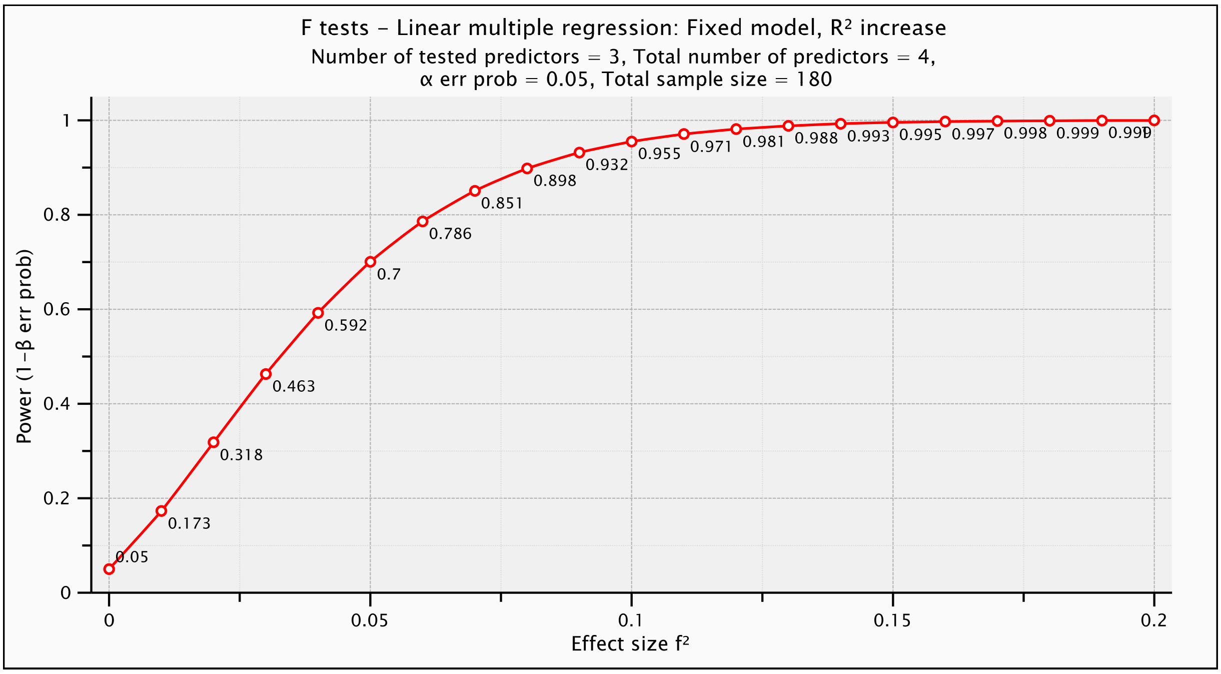

Appendix II

"Problem" Items

Exploratory factor analysis, Cronbach's alpha, and Tucker's congruence analysis reported in this manuscript were calculated using the R package "psych" (Revelle, 2020). For the exploratory factor analysis, we used the default settings: minimum residual solution with oblimin transformation.

Table B1. Descriptive Statistics Problem Items.

	**Zwarte Piet**				**Fireworks**				**Meat consumption**			
	Mean	*SD*	*Median*	*Mode*	*Mean*	*SD*	*Median*	*Mode*	*Mean*	*SD*	*Median*	*Mode*
Personal – Problem?	2.51	1.00	2.00	1	3.43	1.75	3.00	3	3.47	1.43	4.00	4
Social – Problem?	1.88	1.00	1.43	1	1.91	1.19	1.43	1	2.05	1.03	1.86	1
Society – Problem?	2.50	1.54	2.00	1	2.11	1.37	1.50	1	2.30	1.35	2.00	1
Opponents – Problem?	2.64	1.75	2.00	1	1.82	1.23	1.00	1	2.21	1.22	2.00	1
Proponents – Problem?	2.02	1.31	1.50	1	2.04	1.42	1.50	1	2.24	1.31	2.00	1

Table B2. Perceived Opinion Differences about the Debate:
Explorative Factor Analysis including *problem* items (two-factor solution).

	Zwarte Piet		Fireworks		Meat consumption	
	F1	F2	F1	F2	F1	F2
Differences, Personal	0.41	0.11	0.03	0.25	0.13	0.10
> Is this a problem for you?	0.22	0.46	0.36	-0.04	0.39	0.12
Differences, Self vs Family	0.83	-0.05	-0.08	0.73	0.11	0.60
> Is this a problem for you?	0.49	0.49	0.74	0.01	0.58	0.17
Differences, Family	0.79	-0.08	0.04	0.67	0.06	0.57
> Is this a problem for you?	0.44	0.52	0.76	0.08	0.76	0.04
Differences, Self vs Friends	0.78	0.07	-0.05	0.83	0.02	0.74
> Is this a problem for you?	0.37	0.61	0.82	0.11	0.82	0.05
Differences, Friends	0.75	0.06	0.08	0.74	0.00	0.76
> Is this a problem for you?	0.40	0.59	0.88	0.06	0.86	0.03
Differences, Friend vs Family	0.80	0.00	0.04	0.75	0.03	0.71
> Is this a problem for you?	0.37	0.58	0.82	0.08	0.84	0.03
Differences, Self vs Groups	0.72	0.10	0.04	0.72	-0.05	0.72
> Is this a problem for you?	0.28	0.70	0.89	0.02	0.78	0.07
Differences, Groups	0.73	0.06	0.16	0.64	0.05	0.69
> Is this a problem for you?	0.26	0.70	0.89	0.01	0.83	-0.02
Differences, Self vs Society	0.38	0.13	-0.07	0.43	0.04	0.55
> Is this a problem for you?	-0.02	0.87	0.84	0.11	0.83	-0.05
Differences, Society	0.33	0.11	-0.16	0.45	-0.05	0.51
> Is this a problem for you?	-0.02	0.87	0.68	0.02	0.71	0.04
Differences, Self vs Proponents	0.78	-0.17	-0.06	0.15	-0.06	0.61
> Is this a problem for you?	0.42	0.53	0.77	0.10	0.69	0.13
Differences Proponents	0.61	0.11	0.27	0.13	0.07	0.46
> Is this a problem for you?	0.27	0.65	0.77	0.09	0.74	0.07
Differences, Self vs Opponents	-0.17	0.27	-0.04	0.32	-0.09	-0.13
> Is this a problem for you?	-0.27	0.88	0.73	0.02	0.75	-0.22
Differences, Opponents	-0.04	0.26	-0.01	0.36	0.16	0.05
> Is this a problem for you?	-0.02	0.75	0.68	0.02	0.81	-0.15
Proportion Explained	0.51	0.49	0.65	0.35	0.63	0.37
Factor correlations	0.41		0.53		0.45	

Note. Grey shading indicates the highest loading for each item.

Table B3. Multiple Linear Regression predicting Felt Ambivalence, including the "problem" items.

	**Zwarte Piet**				**Fireworks**				**Meat consumption**			
	R^2	*F*	*df*		R^2	*F*	*df*		R^2	*F*	*df*	
**Full Model**	0.40	11.31	10, 172		0.38	10.03	10, 164		0.39	11.24	10, 175	
	B	*SE*	*p*	VIF	*B*	*SE*	*p*	VIF	*B*	*SE*	*p*	VIF
Personal	0.20	0.06	<.01	1.78	0.37	0.07	<.01	1.26	0.21	0.05	<.01	1.42
Personal – Problem?	0.04	0.07	0.53	1.91	0.16	0.06	0.01	1.39	0.01	0.05	0.73	1.75
Social	0.24	0.10	0.02	3.05	0.03	0.08	0.75	1.89	0.28	0.08	<.01	2.05
Social – Problem?	-0.30	0.15	0.05	4.92	0.14	0.11	0.21	3.76	-0.003	0.11	0.97	3.11
Societal - Society	0.03	0.07	0.59	1.90	0.03	0.08	0.70	1.43	-0.19	0.06	<.01	1.71
Society – Problem?	0.19	0.10	0.06	4.50	0.05	0.09	0.60	3.18	0.11	0.09	0.20	3.14
Societal - Opponents	0.005	0.06	0.93	1.54	0.01	0.05	0.82	1.70	0.10	0.06	0.09	1.29
Opponents – Problem?	-0.12	0.08	0.14	3.68	0.05	0.09	0.56	3.39	0.18	0.11	0.09	2.37
Societal - Proponents	0.14	0.08	0.08	2.73	-0.05	0.07	0.42	1.30	0.02	0.05	0.71	1.72
Proponents – Problem?	0.15	0.13	0.26	5.32	0.01	0.10	0.89	2.62	-0.07	0.08	0.37	3.83

Note. The collinearity statistic (VIF) indicates that the predictors are correlated with at least one of the other predictors in the model and that the variance of the underlined coefficients is inflated. Problematic VIF values (above 2.5) are underlined.

Appendix III

Additional Variables

Self-reported doubts and/or mixed feelings. We asked participants whether they have (at least some) mixed feelings or doubts about the debate (e.g., Black Pete, Meat Consumption, Fireworks), using a one-item, self-report measure: "I have doubts/mixed feelings about [debate]" on a 7-point Likert scale (from 1 = "No mixed feelings or doubts at all" to 7 = "Very many mixed feelings or doubts"). We measured this item twice, once at the beginning of the survey and once near the end of the survey. This item was used to pre-select participants; Participants that answered "1" or "2" on the first question were not included in the sample. For descriptive statistics, see Table C1.

Opinion (in favour and against). We measured participants' opinions (in favour and against) using a 2-item measure on a 7-point Likert scale (from 1= "not at all" to 7 "a lot"): 1), e.g., "To what extent are you in favour [Zwarte Piet/Meat Consumption/Fireworks]," and 2)"To what extent are you against [Zwarte Piet/Meat Consumption/Fireworks]." For descriptive statistics, see Table C1.

Involvement with the debate. As an exploratory variable, we asked participants whether they felt involved in the debate measured on a 7-point Likert scale (from 1= "Not at all involved" to 7 = "very much involved". For descriptive statistics, see Table C1.

Perceived (un)importance of debate. We measured how important participants perceived the debate using a one-item self-report measure, "The debate is not important for me personally", with a 7-point Liker scale (from 1= "Not at all important" to 7 = "Very much important"). For descriptive statistics, see Table C1.

Self-reported reasons for ambivalence. We asked participants to rank the reason that was most relevant for their ambivalence or mixed feelings in the debate. Participants could choose and rank up to three sources: *"self," "family," "friends," "colleagues," "groups I belong to," "those in favour," "those against," "society," or "other (...)".* Subsequently, participants were asked to explain their ranking in an open question. For further information on the rankings, see Table C2.

Connectedness. We measured how connected participants felt with the different social sources within the context of the societal debate. We used nine items with a 7-point Likert scale

from 1= *"Not at all connected"* to 7 = *"Very connected"*: "In the context of (debate), how connected do you feel with… [Your family; your friends; the groups you belong to; society; Dutch people; those in favour; those against; others that are ambivalent]". We explored if these items correlated with the levels of felt ambivalence, see Table C3.

Table C1. Descriptive Statistics Additional Measures

	Zwarte Piet			Fireworks			Meat consumption		
	Mean	*SD*	*Median*	*Mean*	*SD*	*Median*	*Mean*	*SD*	*Median*
Mixed Feelings T1	4.80	1.21	5	5.19	1.14	5	4.82	1.13	5
Mixed Feelings T2	3.24	1.74	3	3.51	1.83	4	4.30	1.53	5
Extent in Favour	5.22	1.60	5	3.40	1.80	3	4.21	1.35	4
Extent Against	2.59	1.62	2	4.65	1.78	5	3.73	1.42	4
Perceived (un)importance	3.31	1.77	3	3.61	1.87	4	3.93	1.59	4
Perceived Involvement	3.30	1.74	3	3.67	1.73	4	4.03	1.55	4

Table C2. Summary of Rank Order Question: *Reasons for Ambivalence*

	Zwarte Piet					Fireworks					Meat consumption				
	(N=184)		Ranking			(N=181)		Ranking			(N=187)		Ranking		
	Freq.	*% of N*	#1	#2	#3	Freq.	*% of N*	#1	#2	#3	Freq.	*% of N*	#1	#2	#3
Self	48	*26.09*	33	8	1	74	*40.88*	58	10	6	125	*66.84*	99	10	16
Social															
Family	28	*15.22*	8	7	13	36	*19.89*	10	17	9	44	*23.53*	13	18	13
Friends	13	*7.07*	3	4	6	27	*14.92*	3	15	9	38	*20.32*	2	22	14
Colleagues	15	*8.15*	5	3	7	6	*3.32*		1	5	4	*2.14*	2	1	1
Groups you belong to	25	*13.59*	5	10	10	18	*9.94*	5	6	7	15	*8.02*	3	3	9
Societal															
Society in general	120	*65.22*	54	48	18	105	*58.01*	45	32	28	91	*48.66*	18	48	25
Proponents	61	*33.15*	14	29	18	79	*43.65*	21	38	20	35	*18.72*	9	16	10
Opponents	124	*67.39*	56	42	26	59	*32.60*	15	18	26	81	*43.32*	23	25	33
Other…	10	*5.43*	2	1	7	12	*6.63*	3	4	5	15	*8.02*	5	6	4

Note. All participants could select up to three reasons for their ambivalence and had the opportunity to explain their ratings.

Table C3. Connectedness: Descriptive Statistics and Correlations with Ambivalence

Connected with…		**Zwarte Piet** Ambivalence	**Fireworks** Ambivalence	**Meat consumption** Ambivalence
Family	Mean*(SD)*	3.68*(1.17)*	3.66*(1.31)*	3.45*(1.17)*
	Pearson's r	-0.07	-0.07	-0.01
	95% CI	*[-0.21, 0.08]*	*[-0.21, 0.08]*	*[-0.16, 0.13]*
Friends	Mean*(SD)*	3.57*(1.14)*	3.48*(1.25)*	3.16*(1.11)*
	Pearson's r	-0.03	0.02	0.05
	95% CI	*[-0.18, 0.11]*	*[-0.12, 0.17]*	*[-0.09, 0.19]*
Groups	Mean*(SD)*	3.26*(1.09)*	3.01*(1.21)*	2.69*(1.11)*
	Pearson's r	-0.04	0.01	-0.05
	95% CI	*[-0.19, 0.10]*	*[-0.13, 0.16]*	*[-0.19, 0.09]*
Society	Mean*(SD)*	3.15*(1.06)*	3.13*(1.14)*	3.05*(1.00)*
	Pearson's r	-0.06	0.14	0.05
	95% CI	*[-0.21, 0.08]*	*[-0.01, 0.28]*	*[-0.10, 0.19]*
Opponents	Mean*(SD)*	2.33*(1.18)*	3.33*(1.25)*	2.99*(1.17)*
	Pearson's r	0.35 ***	0.06	0.15 *
	95% CI	*[0.22, 0.47]*	*[-0.08, 0.21]*	*[-0.23, 0.29]*
Proponents	Mean*(SD)*	3.42*(1.22)*	2.63*(1.13)*	3.09*(1.08)*
	Pearson's r	-0.19 *	0.16 *	-0.09
	95% CI	*[-0.32, -0.04]*	*[0.01, 0.30]*	*[-0.23, 0.06]*
The Netherlands	Mean*(SD)*	3.40*(1.06)*	3.30*(1.12)*	3.05*(0.97)*
	Pearson's r	0.13	0.02	0.10
	95% CI	*[-0.01, 0.27]*	*[-0.13, 0.16]*	*[-0.04, 0.24]*
Other "Ambivalents"	Mean*(SD)*	3.05*(1.17)*	3.12*(1.10)*	3.30*(1.02)*
	Pearson's r	0.51 ***	0.39 ***	0.32 ***
	95% CI	*[0.40, 0.61]*	*[0.26, 0.51]*	*[0.18, 0.44]*

* $p < .05$, ** $p < .01$, *** $p < .001$.

Note. The different connectedness scores were mostly uncorrelated with participants' felt ambivalence, with one consistent exception for connectedness with *other ambivalents*.
